# Sentinel node biopsy using blue dye and technetium^99^ in advanced gastric cancer: anatomical drainage and clinical application

**DOI:** 10.1590/1414-431X20165341

**Published:** 2016-07-11

**Authors:** F.A.V. Santos, A.P. Drummond-Lage, M.A. Rodrigues, M.A. Cabral, M.S. Pedrosa, H. Braga, A.J.A. Wainstein

**Affiliations:** 1 Departamento de Cirurgia, Faculdade de Medicina, Universidade Federal de Minas Gerais, Belo Horizonte, MG, Brasil; 2 Instituto de Pós Graduação, Faculdade de Ciências Médicas de Minas Gerais, Belo Horizonte, MG, Brasil

**Keywords:** Gastric carcinoma, Sentinel lymph node, Biopsy, Stage

## Abstract

Lymph node metastases are an independent prognosis factor in gastric carcinoma (GC) patients. Radical lymphadenectomy can improve survival but it can also increase surgical morbidity. As a principle, sentinel node (SN) navigation surgery can avoid unnecessary lymphadenectomy without compromising prognosis. In this pilot study, 24 patients with untreated GC were initially screened for SN navigation surgery, of which 12 were eligible. Five patients had T2 tumors, 5 had T3 tumors and 2 had T1 tumors. In 33% of cases, tumor diameter was greater than 5.0 cm. Three hundred and eighty-seven lymph nodes were excised with a median of 32.3 per patient. The SN navigation surgery was feasible in all patients, with a median of 4.5 SNs per patient. The detection success rate was 100%. All the SNs were located in N1 and N2 nodal level. In 70.9% of cases, the SNs were located at lymphatic chains 6 and 7. The SN sensitivity for nodal staging was 91.6%, with 8.3% of false negative. In 4 patients who were initially staged as N0, the SNs were submitted to multisection analyses and immunohistochemistry, confirming the N0 stage, without micrometastases. In one case initially staged as negative for nodal metastases based on SN analyses, metastases in lymph nodes other than SN were found, resulting in a 20% skip metastases incidence. This surgery is a reproducible procedure with 100% detection rate of SN. Tumor size, GC location and obesity were factors that imposed some limitations regarding SN identification. Results from nodal multisection histology and immunohistochemistry analysis did not change initial nodal staging.

## Introduction

Gastric carcinoma (GC) remains the 4th most common cancer and the 2nd cause of cancer-related deaths worldwide (1), responsible for more than 738,000 deaths every year (2). The chances for a long remission or cure rely mainly on surgical resection, which has changed over the years, especially regarding radical lymphadenectomy procedure (3). Surgical radicality has increased and, consequently, the chances of cure. However, radical lymphadenectomy has also been responsible for increased morbidity (3). Studies concerning tumors like melanoma and breast cancer have used results of radical lymph node dissection more as a marker of prognosis than an opportunity to increase the radicality of surgery and influence overall survival (4,5).

Aiming at decreasing surgical morbidity without compromising surgical outcome, sentinel lymph node (SN) biopsy has been evaluated in patients with GC (6). SNs are the first nodes to receive lymphatic drainage from the primary tumor and can be single or multiple. SN detection allows a more detailed and accurate histological analysis, reducing the possibility of understaging lymph node involvement (7,8). Dissection of the SN is the standard of care for patients with melanoma and breast cancer (7). It is an attractive and promising technique that could also benefit patients with cancers of the digestive tract. SN biopsy is associated with a low morbidity and absence of mortality (9).

Even with the use of sophisticated methods such as spiral computed tomography and endoscopic ultrasound, the preoperative staging of patients with GC may be inaccurate, especially regarding the presence of lymph node metastases (10). As the numbers of GC diagnosed at an early stage have increased, more patients have been considered candidates for endoscopic treatment, which does not determine the stage of lymphatic metastatic disease (11 12–13). In these patients, SN identification would allow more accurate histological evaluation of lymph node involvement, improving the results of endoscopic treatment. However, lymph node metastases in GC do not always follow a linear pattern of lymphatic drainage, and interlocking lymphatic vessels can be responsible for the occurrence of skip metastases (14). SN biopsy results could be used to modify and adapt the lymphadenectomy to each particular case, reducing morbidity and maintaining the effectiveness of the surgical procedure.

This study aimed to evaluate the feasibility of SN biopsy in lymph node staging in patients with gastric cancer, based on preoperative injection of patent blue dye and technetium^99^ (Tc^99^) into the subserosal gastric layer.

## Material and Methods

This was a prospective, descriptive study, comprising patients who underwent curative surgical resection for gastric cancer in a 1-year period at the Hospital da Universidade Federal de Minas Gerais, in Belo Horizonte, MG, Brazil. All patients were informed about the study and signed an informed consent. The study was previously approved by the Universidade Federal de Minas Gerais Ethics Committee.

A total of 24 patients were approached to participate in this study, but 12 were excluded because of benign peptic ulcer (n=1), carcinomatosis and invasion of other organs (n=7), unavailability of nuclear medicine (n=2), and disagreement with the informed consent (n=2). The inclusion criteria were as follows: patients with histologically confirmed GC that were going to be submitted to radical gastrectomy according to the guidelines of the Japanese Gastric Cancer Association (JGCA) (15). Patients with synchronous multicentric tumors, previous gastric surgery, and metastatic disease were excluded.

**Figure 1:**
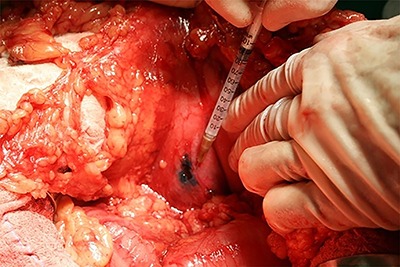
Preoperative Tc^99^ injection into the lesser curvature, of the posterior gastric wall, around the tumor. Note the blue discoloration as a result of the injected patent blue.

**Figure 2:**
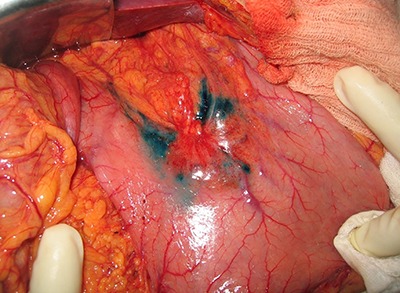
Final aspect after the blue dye injection. Observe the blue discoloration as a result of patent blue injected around the tumor (perioperative aspect).

**Figure 3:**
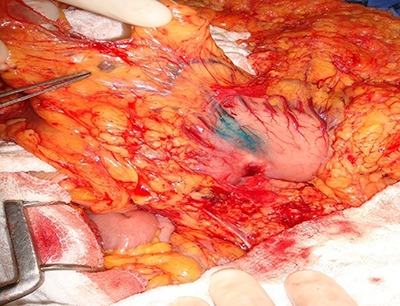
Perioperative sentinel node identification by patent blue (tip of tweezers). The tumor is located in the posterior gastric wall.

Patent blue dye (Guerbet, France) (0.2 mL) was injected into the subserosa gastric layer at four spots around the tumor (Figure 1), intercalated with Tc^99^ (0.2 mL) (National Nuclear Energy Agency, Brazil) at each spot (Figure 2). In the perioperative period, the SN was indicated by a blue discoloration and/or by radioactivity (Figure 3). Blue dyed lymph nodes were marked with a blue Prolene® (Ethicon, USA) suture (3.0) and the radioactive lymph nodes (Tc^99^) with a black silk suture (3.0). At the end of the surgical procedure, the surgical specimen was dissected, and all SNs were classified according to the related lymphatic chain.

SN samples were histologically analyzed on hematoxylin-eosin (HE) sections by a single pathologist. When negative for metastases, the nodes were submitted to multisection analyses and immunohistochemistry (IHC) for cytokeratin.

## Results

Total gastrectomy was performed in 11 patients and a distal subtotal gastrectomy in 1 patient, always associated with D2 lymphadenectomy. According to the Lauren classification, tumors were histologically classified as intestinal type in 8 patients and as diffuse type in 4 patients. The average tumor diameter was 4.4 cm, ranging from 1.6 to 10 cm. Four patients (33%) had tumors larger than 5 cm in diameter. Five patients had tumors located in the proximal third of the stomach, 5 had tumors located in the middle third, and 2 had distal tumors. Five tumors extended to the serosal layer (T3), 1 reached the subserosa (T2b), 4 infiltrated the muscularis propria (T2a), 1 invaded the submucosa (T1b), and another invaded the mucosa (T1a) (Table 1). This classification reflects a common situation found in countries like Brazil where the majority of patients have gastric cancer diagnosed at late stages. Despite this, we applied the SN technique in an effort to optimize staging and consequently, the treatment of these patients.

**Table:**
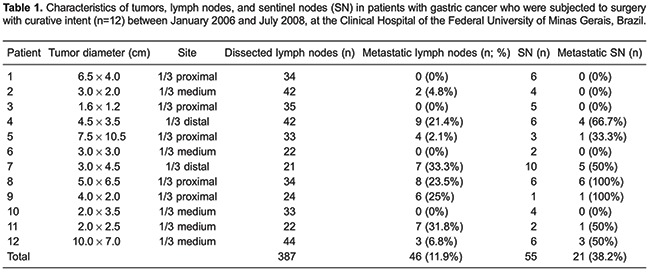

A total of 387 lymph nodes were dissected (a mean of 32.2 lymph nodes per patient), and 46 (11.9%) from 8 patients were metastatic. In 4 of these patients, the tumor had grown into the serosal layer and in the other 4 patients, it involved the muscularis propria. Of the 4 patients who had no lymph node metastases, 1 had a tumor extending to the serosa, 1 to the subserosa, 1 to the submucosa, and the other had a tumor restricted to the mucosal layer (Table 2).

In three cases, there was disagreement between the lymph node staging according to the JGCA and to the American Joint Committee on Cancer (AJCC). Based on the AJCC, the number of lymph node metastases was equal to or less than six (N1), while according to JGCA, extraperigastric lymph node metastases were staged as N2 (Table 3).

**Table:**
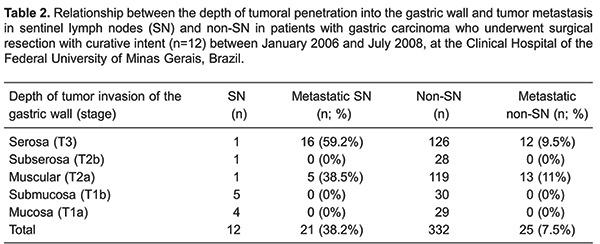

**Table:**
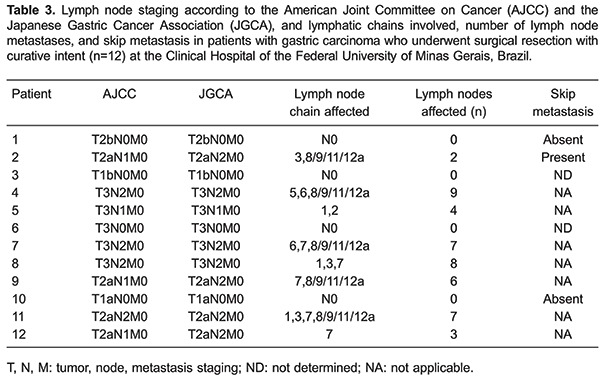

SN biopsy was feasible in all 12 cases with a detection rate of 100% using the combination of patent blue and Tc^99^. No complications related to SN biopsy were observed. In one case, SN biopsy with Tc^99^ was hampered by the impossibility of measuring the radioactivity of the lymph nodes due to contamination of the operative field by the accidental injection of Tc^99^ in the stomach lumen. Thus, SN detection rate using Tc^99^ as the main marker was 83%. In 55 SNs, 13 were detected using patent blue alone, six were identified using Tc^99^ alone, and 36 using both markers, resulting in an 89.1% sensitivity in SN identification.

The number of SNs per patient ranged from 1 to 10, with a mean of 4.5 lymph nodes. In 3 patients whose tumors infiltrated the muscularis propria or serosa, SNs were located exclusively in group 2 lymph nodes (N2, chain 7). In 2 patients who had tumors infiltrating the mucosal layer and the muscularis propria, SNs were located exclusively in group 1 lymph nodes (N1, chains 3, 4, and 6). Around 70% of SNs were distributed in chains 7 (27 lymph nodes) and 6 (12 lymph nodes) (Table 4). Table 5 shows the distribution of SNs and their respective extraperigastric and perigastric lymph node chains.

**Table:**
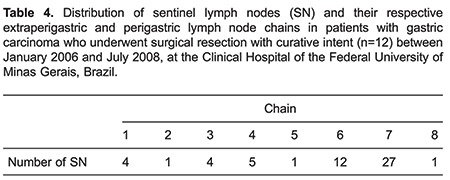

**Table:**
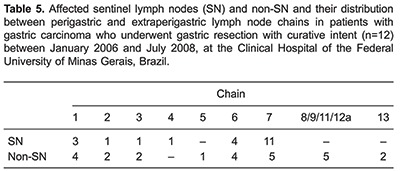

Eight cases were classified as intestinal type according to Lauren's classification. As shown in Tables 6 and 7, the number of SNs and non-SNs, as well as the presence or absence of metastatic lymph nodes between the intestinal and diffuse types were discordant.

**Table:**
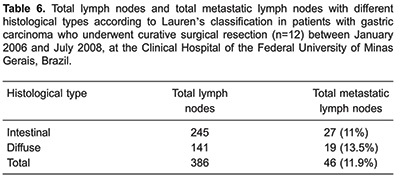

**Table:**
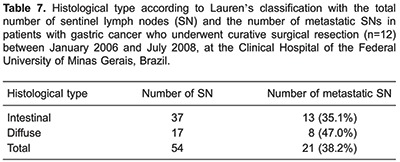

HE and multi-dissection analyses showed that in five cases SNs were not metastatic, although IHC data of two of these cases were inaccurate and therefore not included in the statistical analysis. In the other three cases, SNs were negative for the presence of micrometastasis, as demonstrated by both multi-dissection and IHC analyses.

Five patients presented skip metastasis (20%), defined as cases in which SNs were free of tumor in HE, multi-dissection, and IHC analyses, but non-SNs were affected. Most of these patients had tumor invading the muscularis propria, involving the lesser curvature of the middle third of the stomach, with a diameter over 2.0 cm, classified as intestinal type according to Lauren's criteria, and with lymphatic invasion. In these cases, we found four SNs marked by patent blue and Tc^99^, which were located in the lymphatic chain 7, the hepatic artery chain. IHC analysis confirmed the results obtained by the routine histological evaluation (HE) and nodal multi-dissection.

## Discussion

Most GCs are adenocarcinomas, in which lymphatic spread can occur even in early stages of the disease and is found in a high percentage of patients.

Of the 24 patients initially enrolled in this study, 7 (34%) were excluded because they had advanced tumors, some with deep invasion of gastric wall and dissemination into the peritoneal cavity or invasion of contiguous organs. Indeed, it has been shown that, especially in the Western world, most cases of GC are diagnosed in advanced stages and often with a metastatic disease at diagnosis (2).

The average tumor diameter observed in the current study was greater than that reported in the literature on the application of SN biopsy in gastric cancer (16). Usually, a tumor diameter equal to or greater than 5.0 cm is excluded from the analysis because of the high incidence of lymph node metastasis and destruction of the natural routes of perigastric lymphatic drainage. This impairs the identification of SNs and justifies radical lymphadenectomy (17). We did not exclude these cases from our study because our main purpose was to evaluate the applicability and feasibility of SN biopsy even in advanced GC. The inclusion of these cases contributed to identifying the technical difficulties in the implementation of the method and in establishing measures to facilitate its application in advanced stage GC.

In this study, SN biopsy was based on palpation and visual identification of the tumor, perioperative injection of the markers and direct observation of their migration through the lymphatic chains. We observed that the very small and the large lesions interfered with SN biopsy. When tumors were very small and difficult to palpate, nonspecific thickening of gastric folds could easily be mistaken for tumors, impairing the correct identification of SNs by inducing the injection of the markers without including the whole tumor perimeter. In these cases, endoscopic injection of the markers would be more appropriate as it allows direct visualization of the tumor limits. On the other hand, the difficulty in identifying the tumor boundaries and in some cases, the gastric wall limits of bulky tumors, especially those invading the serosa and inducing retraction of adjacent tissues, may have hindered the correct injection of tracers.

We also observed that the location of the tumor in the stomach can hinder the identification of SNs. Proximal lesions located at the lesser curvature and extending to the posterior wall of the stomach hampered the access for injection of the markers, especially when the patient is obese, misguiding the injection of tracers into the gastric wall. To avoid this limitation, some authors have recommended endoscopic injection of markers (18). In this study, we adapted the procedure to the reality of Brazil, making it simpler and easier, minimizing patient manipulation one day before surgery and endoscopy costs, as previously reported (12).

Some studies have postulated that the volume of tracers injected is directly related to the number of SNs identified. A total volume exceeding 4 mL may lead to the identification of high numbers of SNs, decreasing the sensitivity of the method. Therefore, total volumes greater than 2 mL are not recommended (19). In this study, tracers were injected into each spot of the gastric wall in a volume of 0.2 mL. The total volume injected was 1.6 mL, consisting of 0.8 mL of Tc^99^ and 0.8 mL of patent blue. The injected volume was standardized and therefore we could not assess the ratio between volume of the injected marker and the number of SNs.

In agreement with data demonstrating the rapid migration of tracers through the lymphatic system (13), we observed that the time between the injection of the tracer and its detection in the SN was 5 to 10 min. These findings emphasize that dyes should be injected in the perioperative period.

Using patent blue and Tc^99^ combined, we could identify SNs in all cases. We detected an average of 4.5 SNs per patient, a number consistent with that reported in the literature (20). All SNs were located at N1 and N2 lymph node stations. The sensitivity of SN identification for metastasis diagnosis was 91.6%, with a false negative rate of 8.3%. These results are in agreement with most studies showing sensitivity and false negative results for the combined SN biopsy of approximately 80 and 8%, respectively (21–23).

We could not precisely identify the SN by measuring the radiation emitted by Tc^99^ in one case, due to contamination of the operative field. In cases of accidental injection of Tc^99^ into an organ and to reduce the technical difficulties caused by the interference of radiation emitted from Tc^99^ injected into the gastric wall, some authors have suggested to perform SN biopsy in the immediate postoperative period (24).

Approximately 71% of SNs identified in this study were located at the infrapyloric and gastric artery lymphatic chains (chains number 6 and 7). In most cases (58.3%), SNs were located in N1 and N2 levels. These findings confirm the role of perigastric lymphatic chains and the chain along the left gastric artery as a route for tumor drainage (6). These results are different than those described in the literature, in which SNs were distributed exclusively in N1 lymph node level in 62% of cases and in N2 level in 13% of cases (25,26). This may be related to the advanced stage of the tumors analyzed in this study, as well as to the reduced sample size.

Multisection and HE analysis revealed the absence of micrometastases in the patients. This result may be related to the small number of cases studied, especially considering that two records in which the initial lymph node staging was N0 were excluded from the study. It has been shown that both IHC and RT-PCR have greater sensitivity for detection of micrometastases than HE (27,28). Indeed, a previous study reported that the incidence of micrometastases detected by HE and IHC was 8.2% and 13.7%, respectively. Metastases not identified by IHC were found in 23% of patients when RT-PCR was applied (27).

The final lymph node staging N0 (absence of metastases) was found in 4 patients. Metastasis was observed in 8 patients, and in all of them the tumors were advanced (T2a and T3). Of these patients, 7 had SNs affected. In the other patient who had a bulky tumor invading the muscular layer, SNs were free of metastasis, but the non-SNs were affected, characterizing a case of skip metastasis. Confirming the tendency reported by other studies (29), we found skip metastasis in the same lymphatic chain where the SN was located (gastric artery lymphatics). Skip metastasis may be related to an anomalous route of lymphatic drainage, to the depth of tumor invasion in the gastric wall, or to the occlusion of the lymphatic drainage route by tumor embolism (29).

The SN procedure is a promising technique for the staging of gastric cancers and also in the establishment of precise staging in neoadjuvant treatment protocols, like in the Magic Trial (30). Patients initially identified as N1 or higher could be corrected to N0 after analyzing surgical specimens from the neoadjuvant treatment, influencing the real prognosis and even future systemic treatments. In this study, we showed that SN biopsy is technically feasible in patients with GC. For cases in which a single marker should be used in SN biopsy, patent blue is the most suitable because of its low cost, applicability, and reproducibility. The technique was highly sensitive in identifying node metastases, although false negative results and skip metastases restrict the application of SN biopsy as a single method for lymphatic staging.
